# Neuronal gamma-aminobutyric acid (GABA) type A receptors undergo cognate ligand chaperoning in the endoplasmic reticulum by endogenous GABA

**DOI:** 10.3389/fncel.2015.00188

**Published:** 2015-05-18

**Authors:** Ping Wang, Randa S. Eshaq, Charles K. Meshul, Cynthia Moore, Rebecca L. Hood, Nancy J. Leidenheimer

**Affiliations:** ^1^Department of Biochemistry and Molecular Biology, Louisiana State University, Health Sciences Center-ShreveportShreveport, LA, USA; ^2^Veterans Hospital Portland/Research Services/Neurocytology Laboratory and Department of Behavioral Neuroscience, Oregon Health & Science UniversityPortland, OR, USA

**Keywords:** GABA_A_ receptor, GABA, GABA transaminase, ligand chaperone, endoplasmic reticulum, calnexin

## Abstract

GABA_A_ receptors mediate fast inhibitory neurotransmission in the brain. Dysfunction of these receptors is associated with various psychiatric/neurological disorders and drugs targeting this receptor are widely used therapeutic agents. Both the efficacy and plasticity of GABA_A_ receptor-mediated neurotransmission depends on the number of surface GABA_A_ receptors. An understudied aspect of receptor cell surface expression is the post-translational regulation of receptor biogenesis within the endoplasmic reticulum (ER). We have previously shown that exogenous GABA can act as a ligand chaperone of recombinant GABA_A_ receptors in the early secretory pathway leading us to now investigate whether endogenous GABA facilitates the biogenesis of GABA_A_ receptors in primary cerebral cortical cultures. In immunofluorescence labeling experiments, we have determined that neurons expressing surface GABA_A_ receptors contain both GABA and its degradative enzyme GABA transaminase (GABA-T). Treatment of neurons with GABA-T inhibitors, a treatment known to increase intracellular GABA levels, decreases the interaction of the receptor with the ER quality control protein calnexin, concomittantly increasing receptor forward-trafficking and plasma membrane insertion. The effect of GABA-T inhibition on the receptor/calnexin interaction is not due to the activation of surface GABA_A_ or GABA_B_ receptors. Consistent with our hypothesis that GABA acts as a cognate ligand chaperone in the ER, immunogold-labeling of rodent brain slices reveals the presence of GABA within the rough ER. The density of this labeling is similar to that present in mitochondria, the organelle in which GABA is degraded. Lastly, the effect of GABA-T inhibition on the receptor/calnexin interaction was prevented by pretreatment with a GABA transporter inhibitor. Together, these data indicate that endogenous GABA acts in the rough ER as a cognate ligand chaperone to facilitate the biogenesis of neuronal GABA_A_ receptors.

## Introduction

The neurotransmitter γ-aminobutyric acid (GABA) is the main inhibitory neurotransmitter in the central nervous system. Approximately 30% of synapses in the brain contain GABA_A_ receptors (Nutt, 2006), a subtype of GABA receptor that mediates fast inhibitory neurotransmission. Upon binding GABA, an integral chloride channel within the receptor is gated, allowing chloride influx thus leading to membrane hyperpolarization and neuronal inhibition. The GABA_A_ receptor is associated with a variety of psychiatric (anxiety, schizophrenia) and neurological (epilepsy, insomnia) disorders (Möhler, 2006; Charych et al., 2009). Importantly, the receptor is the target of several classes of widely used therapeutic agents including benzodiazepines, barbiturates and anesthetics (Whiting, 2003).

GABA_A_ receptors belong to the Cys-loop ligand-gated ion channel superfamily, whose other members include the nicotinic acetylcholine, glycine and serotonin type 3 receptors (Olsen and Sieghart, 2008). Cys-loop ligand-gated ion channels are pentameric in structure, with each subunit possessing a large extracellular N-terminus, four membrane-spanning domains (M1–M4), a large cytoplasmic loop between M3 and M4, and a short extracellular C-terminus. Although 19 GABA_A_ receptor subunits have been identified only a limited number of receptor subtypes exist based on developmental/tissue expression and assembly residues that specify oligomerization interfaces (Olsen and Sieghart, 2009). The various receptor subtypes display distinct functional characteristics, cellular/subcellular distributions, pharmacology, protein/protein interactions and posttranslational modifications. The predominant receptor subtype in brain is composed of α1β2γ2 subunits, with two copies of α1 and β2 subunits (McKernan and Whiting, 1996). Two GABA binding sites are present on each pentamer, with the GABA binding pocket formed at the αβ subunit interfaces by subunit N-terminal regions (Amin and Weiss, 1993). Of particular relevance to the present study, the receptor topology places the GABA binding pocket within the lumen of the RER during receptor biogenesis.

Given that the concentration of synaptically-released GABA is often saturating for postsynaptic receptors, the efficacy and plasticity of fast GABAergic neurotransmission is largely determined by the number of GABA_A_ receptors expressed at the cell surface (Otis et al., 1994; Nusser et al., 1997). Recognizing this, numerous studies have been directed toward understanding the processes that regulate GABA_A_ receptor cell surface expression including exocytosis, lateral diffusion, surface stabilization, endocytosis, recycling and degradation (reviewed in Luscher et al., 2011). Despite extensive investigation of these processes, little is known regarding post-translational mechanisms in the RER that regulate GABA_A_ receptor surface levels.

Emerging evidence suggests that the RER functions as a reservoir for integral membrane proteins from which inactive nascent pools can be repartitioned into functional pools (Breitwieser, 2014; Leidenheimer and Ryder, 2014). Consistent with this concept, pharmacological chaperones act post-translationally in the RER to facilitate the biogenesis, and subsequent trafficking and function of their target proteins (Bernier et al., 2004; Ulloa-Aguirre and Michael Conn, 2011; Breitwieser, 2014; Leidenheimer and Ryder, 2014; Srinivasan et al., 2014). While the majority of studies investigating pharmacological chaperones have examined their ability to rescue the biogenesis of RER-retained, disease-causative mutants, pharmacological chaperones also promote the biogenesis of inefficiently processed wild type proteins such as δopioid (Petäjä-Repo et al., 2002; Chen and Liu-Chen, 2009), β-adrenergic (Kobayashi et al., 2009), dopamine D4 (Van Craenenbroeck et al., 2005), and nicotinic acetylcholine (Kuryatov et al., 2005; Sallette et al., 2005; Srinivasan et al., 2011) receptors. The ability of exogenous ligands to act as ligand chaperones in the RER raises the question of whether endogenous ligands may function as “cognate ligand chaperones” to regulate the biogenesis of their receptors. It has been suggested that such regulation may occur for choline/nicotinic acetylcholine receptors (Sallette et al., 2005), growth hormone/growth hormone receptors (van den Eijnden and Strous, 2007), GABA/GABA_A_ receptors (Eshaq et al., 2010) and adenosine/A1-adenosine receptors (Kusek et al., 2015). Furthermore, based on the observation that glutamate receptors with mutated glutamate binding domains are retained in the RER, it has been suggested that glutamate occupancy of nascent glutamate receptors may be required for efficient biogenesis of NMDA, kainate and AMPA glutamate receptors (Grunwald and Kaplan, 2003; Mah et al., 2005; Valluru et al., 2005; Fleck, 2006; Penn et al., 2008; Coleman et al., 2009, 2010; Gill et al., 2009; Kenny et al., 2009; She et al., 2012). The present work extends our previous findings that exogenous GABA acts as a ligand chaperone of recombinant GABA_A_ receptors (Eshaq et al., 2010) by investigating whether native GABA_A_ receptors in primary neuronal cultures undergo cognate ligand chaperoning by endogenous GABA.

## Methods

### Drugs

Vigabatrin, picrotoxin and valproic acid were obtained from Sigma. NCC 05-2090 HCl and R-(+) baclofen HCl were obtained from Tocris and Santa Cruz Biotechnology, respectively.

### Primary Cortical Neuronal Cultures

Timed-pregnant Sprague-Dawley rats (Harlan or Charles River) were singly housed in an AALACC approved facility. Primary cerebral cortical neuronal cultures were prepared from E18 embryos. Experimental procedures were approved by the Animal Care and Use Committee of LSU Health Sciences Center-Shreveport in accordance with NIH guidelines. To prepare primary cerebral cortical cultures, cerebral cortices were dissected from E18 brains, minced in calcium/magnesium free Hank’s balanced salt solution (HBSS) (Invitrogen), triturated in HBSS containing 0.05 mg/ml deoxyribonuclease I (Sigma), and incubated for 15 min in trypsin-EDTA solution (Invitrogen) at 37°C. Dissociated neurons were gently pelleted, resuspended in Neurobasal medium (Invitrogen) containing 10% heat-inactivated fetal bovine serum (FBS, Atlanta Biologicals), filtered through sterile gauze, supplemented with 10% FBS and plated on 35 mm poly-D-lysine-coated plates at a density of either 2,500,000 cells/plate (high-density) or 250,000 cells/plate (low-density, glass-bottom insert dishes, Mattek) and cultured at 37°C in a humidified atmosphere of 95% air and 5% CO_2_. For high-density neurons, at days *in vitro* (DIV) 3, 10 μM β-cytosine arabinoside (ARC, Sigma) was added to the culture medium to inhibit glial cell proliferation. For low-density cultures, medium was replaced 3 h post-plating with serum-free Neurobasal medium supplemented with B-27 (Invitrogen). For low-density neurons, a quarter media change with glial-conditioned serum-free medium supplemented with 10 μM ARC was performed at DIV 3. Low-density cultures were fed thereafter once per week with glial-conditioned serum-free medium. Neuronal cultures were used for experiments between DIV 12–15.

### Immunofluorescence Labeling and Confocal Microscopy

#### Double-Labeling Experiments for Detection of GABA or GABA-T in Cells Expressing Surface GABA_A_ Receptors

Living low-density neurons were incubated with a mouse monoclonal anti-β2/3 subunit antibody (1:100, Millipore, clone 62–3G1) for 1.5 h at room temperature. Cells were then fixed in 4% paraformaldehyde, permeabilized with Triton X-100 (5%) and incubated with a highly-adsorbed Alexa 594-conjugated donkey anti-mouse antibody (1:1000, Invitrogen). Neurons were then blocked with 10% FBS and incubated overnight with either a rabbit polyclonal anti-GABA (1:1000, Sigma) or anti-GABA transaminase (1:100, 4-aminobutyrate aminotransferase ABAT; Proteintech) antibody. An Alexa 488-conjugated donkey anti-rabbit antibody (1:1000, Invitrogen) was used for the detection of primary antibodies.

#### Receptor Insertion Protocol

Living low-density neurons were incubated with a mouse monoclonal anti-β2/3 subunit antibody (1:100, Millipore, clone 62–3G1) for 1.5 h at room temperature. Room temperature incubations allow receptor insertion into the plasma membrane (Lu et al., 2001; Sun et al., 2005) but not endocytosis (Machu et al., 2006) and Figure 4C. Cells were then fixed (4% paraformaldehyde), permeabilized (5% Triton X-100) and incubated with a highly-adsorbed Alexa 594-conjugated donkey anti-mouse antibody (1:1000, Invitrogen).

#### Receptor Endocytosis Protocol

Living neuronal cultures were incubated with mouse monoclonal anti-β2/3 subunit antibody for 1.5 h at either 37°C or room temperature. Neurons were then placed on ice and incubated in a donkey anti-mouse secondary antibody (1:1000) for 1 h. to detect surface receptors that remained on the cell surface during the primary antibody labeling step. Cells were then fixed in 4% paraformaldehyde and permeabilized with 5% Triton X-100. To detect receptors that had undergone endocytosis during the primary antibody labeling period, internalized receptor was detected with a donkey anti-mouse secondary antibody conjugated to Alexa 594.

#### Image Acquisition and Analysis

Images for the above experiments were acquired using a Leica DMI 6000 CS inverted laser-scanning confocal microscope (argon/krypton laser) with a Plan Apo 63x/glycerol immersion objective. The laser intensity, photomultiplier gain, and iris were optimized for each experiment and kept constant within that experiment. A Z-series of twenty optical sections (0.2 μm/section) was collected to capture three dimensional information and the series was reconstructed to yield images. For the insertion experiments, the images were analyzed using NIH ImageJ 1.43 software. For each image, the anti-β2/3 subunit fluorescence signal was analyzed for fluorescence intensity, number of puncta and puncta size (range 0.1–2.5 μm). Each experiment was performed in triplicate with at least two images acquired for each triplicate dish to yield six images for each experimental condition per experiment. All quantified data were acquired and analyzed by an investigator blinded to the experimental treatments.

### Coimmunoprecipitation Experiments

High-density neuronal cultures were treated at 37°C with drugs (vigabatrin, picrotoxin, NCC 05–2090 HCl, baclofen or valproic acid) at concentrations and treatment times indicated in the results section. Following treatment, cells were harvested in RIPA buffer (Tris-HCl 50 mM, pH, 7.4; NaCl 150 mM, 0.5% sodium deoxycholate, 1% NP-40, 0.1% SDS and EDTA 5 mM) containing protease inhibitor cocktail (Roche Diagnostics), sonicated, centrifuged at 16,000 × *g* for 10 min, and the protein concentration of the supernatant determined using a bicinchoninic protein assay kit (Pierce). To immunoprecipitate the GABA_A_ receptor α1 subunit or insulin receptor β chain under nondenaturing conditions, 500 μg of protein lysate was incubated overnight at 4°C with 2 μg of either polyclonal rabbit anti-α1 subunit antibody (α1 N_(1–9)_, generously provided by Dr. Werner Sieghart, Medical University of Vienna), rabbit monoclonal anti-insulin receptor β chain antibody (Cell Signaling Technology) or control IgG (Figure 2E) and 40 μl of protein A Agarose beads (1:1 slurry) (Millipore). Immunoprecipitated samples were pelleted, washed and resuspended in Laemmli sample buffer. Neuronal lysates and immunoprecipitates were each resolved by SDS-PAGE and transferred to polyvinylidene fluoride membranes (Millipore). Membranes were incubated with either polyclonal rabbit GABA_A_ receptor anti-α1 subunit (1:1000, Phosphosolutions), polyclonal rabbit anti-calnexin (1:1000, Enzo Life Sciences), monoclonal mouse anti-actin (Abcam) or rabbit monoclonal anti-insulin receptor β chain (Cell Signaling Technology) antibody overnight at 4°C followed by incubation with the appropriate horseradish peroxidase (HRP)-conjugated secondary antibodies (Jackson ImmunoResearch Laboratories). In order to detect the 50 kDa receptor α1 subunit without interference from the 50 kDa IgG heavy chain of the immunoprecipitating antibody, a light chain-specific secondary antibody was used (Figure 2E). HRP activity was detected by enhanced chemiluminescence (Pierce kit) using film. Film exposure times were adjusted to yield band intensities in the linear range. The integrated density volumes (IDVs, i.e., pixel intensity × mm^2^) of each band was measured using NIH Image J 1.43 software. IDVs obtained from regions adjacent to the bands of interest were used for background subtraction. Lysate data were normalized to actin.

### Glycosidase Digest Experiments

Anti-α1 subunit antibody immunoprecipitations were performed as described above. Immunoprecipitates from each condition (control and VGB-treated) were divided equally into three tubes for either mock-treatment (undigested), or digest with either Endo H or PNGase. Immunoprecipitates were then denatured at 100°C for 3 min and digested for 2 h at 37°C with 0.005 units/μl of Endo-β-N-acetylglucosaminidase H (Endo H, Sigma) in sodium citrate buffer (50 mM sodium citrate, pH = 5.5, 1% Triton X-100, 0.1% SDS, 50 mM β-mercaptoethanol) or 500 units/μl Peptide-N-glycosidase F (PNGase, New England Biolabs) in Tris buffer (50 mM Tris-HCl, pH = 7.4, 1% Triton X-100, 0.1% SDS, 50 mM β-mercaptoethanol). Additional samples were mock digested (no enzyme, i.e., “undigested”). Laemmli buffer was then added to each sample, samples were boiled and subjected to SDS PAGE/Western blotting with an anti-α1 subunit antibody (1:1000, Phosphosolutions) and a light chain-specific, HRP-conjugated secondary antibody.

### DAB and Immunogold GABA Double Labeling and Electron Microscopy

Mice (C57 Bl/6J, males, 12 weeks old, Jackson Labs, Bar Harbor, ME) were used for these experiments. Handling and care of mice was consistent with federal guidelines of the Public Health Service Policy on the Humane Care and Use of Laboratory Animals and this protocol was approved by the Portland VA IACUC. Retrograde tracing to localize glutamate-containing neurons in the motor cortex was performed by biotinylated dextran amine (BDA) injections into the underlying dorsolateral striatum. A 10% solution of 3 kDa BDA (Lysine Fixable: BDA-3000; Life Technologies, Eugene, OR) was prepared in 0.1 M sodium citrate-HCl (pH 3.0), as described by Reiner et al. (2000). Mice were anesthetized with isoflurane and positioned in a stereotaxic frame (Kopf Instruments, Tujunga, CA). The dorsolateral quadrant of the striatum (AP +1.0 mm, ML +2.0 mm, DV −3.0 mm) (Paxinos and Franklin, 2004) was infused with 200 nl of BDA using a Nanoject II (Drummond Scientific, Broomall, PA) apparatus for delivery of the tracer. Mice were euthanized by transcardial perfusion with 2.5% glutaraldehyde/0.5% paraformaldehyde/0.1% picric acid in 0.1 M phosphate buffer (pH 7.3) 1 week after surgery to allow for retrograde transport of the BDA to the overlying motor cortex neurons. Brains were removed, cut in half coronally at the level of the hypothalamus and the rostral half, containing the BDA infused striatum was placed in a microwave tissue processing unit (Pelco BioWave, Ted Pella, Inc., Redding, CA) containing a temperature controlled fixation bath using a thermoelectric recirculating chiller (Pelco Steady Temp Pro, Ted Pella Inc) for a total of 60 min (30 min., 150 watts(W) at 28°C; 15 min., 150 W at 25°C; 15 min., 650 W at 25°C). The brain was then rinsed and left in 0.1 M phosphate buffer (PB) at 4°C for overnight. Following vibratome (Leica Microsystems, Buffalo Grove, Il) sectioning (60 μm) and collection of motor cortex slices [equivalent to: +0.5 to +2.0 mm; (Paxinos and Franklin, 2004), the tissue was then processed in the microwave oven for localization of the BDA tracer using diaminobenzidine (DAB) histochemistry. The tissue was exposed to an Avidin/Biotin enzyme complex (ABC), (Vector Laboratories Burlingame, CA) under constant vacuum, with a temperature limit of 30°C for a total of 15 min (3’ at 150 W; 4’ at 0 W; 3’ at 150 W; 5’ at 150 W). Following one rinse with phosphate buffered saline (PBS) and one with the imidazole buffer (25 mls of 0.2 M imidazole, pH 9 + 79 mls of 0.1 M sodium acetate, in 396 mls water) (1 min. each at 100 W, and temp. limit of 30°C), the tissue was exposed to DAB [5 mg/10 mls of imidazole buffer + 2 μl of 30% H_2_O_2_ just prior to use) for 10 min., constant vacuum, 150 W and a temperature limit of 30°C. This was then followed by 1 final rinse with imidazole buffer and then PBS (1 min. each at 100 W, and temp. limit of 30°C). The tissue was then processed in the microwave oven (Pelco BioWave, Ted Pella, Inc, Redding, CA) and embedded in epoxy as previously described (Schang et al., 2010; Walker et al., 2012). Post-embedding immunogold electron microscopy was performed according to previous methods with modifications (Phend et al., 1992; Meshul et al., 1994; Schang et al., 2010). Thin sections (60–80 nm) were cut using an ultramicrotome (UltracutE, Leica, Microsystems, Buffalo Grove, Il). The GABA antibody (non-affinity purified, rabbit polyclonal; Sigma Chemical Co., St. Louis, MO), as previously characterized (Phend et al., 1992), was diluted 1:250 in TBST pH 7.6 and applied to the thin sections overnight. We previously reported that incubation of this antibody with 3 mM GABA resulted in no immunogold labeling, showing the specificity of the GABA labeling (Meshul et al., 1994). A goat anti-rabbit IgG secondary antibody to detect the primary antibody, tagged with 12 nm gold particles (Jackson ImmunoResearch Laboratories, Inc., West Grove, PA) was diluted 1:25 in TBST pH 8.2 and incubated for 90 min with the thin section. After washing the grids twice in TBST 8.2 and then water, the thin sections were counterstained with uranyl acetate and lead citrate. Photographs of DAB labeled neurons within Layer V of the motor cortex were randomly taken. A total of ten DAB neurons were photographed and gold particles were counted within the same cells for the RER (intraluminal and membrane associated), mitochondria (inside and membrane associated) and cytoplasm (outside of organelles) using ImagePro Plus Imaging program (Media Cybernetics, Rockville, MD). The density of gold particles/μm^2^ was then calculated and compared between the various subcellular compartments.

## Results

### Neurons Expressing Surface GABA_A_ Receptors Contain the Neurotransmitter GABA and its Degradative Enzyme GABA Transaminase

**Figure 1:**
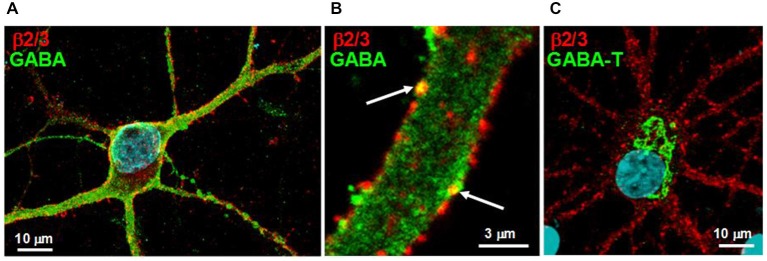
**Neurons expressing surface GABA_A_ receptors contain both the neurotransmitter GABA and its degradative enzyme GABA transaminase**. **(A)** Living low-density neuronal cultures were immunolabeled for surface GABA_A_ receptors using an anti-β2/3 subunit antibody, fixed, permeabilized and immunolabeled for the neurotransmitter GABA. Surface receptors (red) are distributed throughout both the soma and processes, whereas GABA immunoreactivity (green) is observed throughout the neuron but most prominently in the processes. **(B)** An enlarged image of a neuronal process immunolabeled as described in **(A)**. Note the punctate distribution of the receptor, the diffuse cytoplasmic staining of GABA and the colocalization of GABA with some surface receptor puncta (arrows). **(C)** Neuronal cultures were immunolabeled for surface GABA_A_ receptors using an anti-β2/3 subunit antibody, fixed, permeabilized and immunolabeled for GABA transaminase (GABA-T) (green). Note that GABA transaminase immunoreactivity is localized to the cell soma.

For GABA to act as a ligand chaperone, GABA must be present in neurons that express surface GABA_A_ receptors. While GABA would be expected to be present in boutons of GABAergic neurons, we sought to determine whether GABA would be more widely distributed throughout neurons. To determine whether this is the case, immunofluorescence experiments were conducted in which primary cerebrocortical cultures were dually-labeled for the presence of surface GABA_A_ receptors and GABA. To label GABA_A_ receptors, an anti-β2/3 subunit antibody was used that recognizes an extracellular epitope common to both β2 and β3 subunits. Of the three β subunit isoforms, mature cortical neurons contain predominantly β2 and β3 subunits (Pirker et al., 2000), therefore an anti-β2/3 subunit antibody would presumably detect the majority of GABA_A_ receptors present in the cultures. For these experiments, surface anti-β2/3 subunit labeling was performed to ensure the measurement of pentameric receptors. Consistent with the literature (Craig et al., 1994), surface anti-β2/3 subunit staining was observed on the cell soma and throughout the processes (Figures 1A,B). Importantly, anti-GABA staining was observed throughout the soma and proximal dendrites of neurons expressing surface GABA_A_ receptors (Figures 1A,B). Approximately 55% of β2/3 immunopositive cells were immunopositive for GABA, with the intensity of the GABA signal varying between cells. The anti-GABA signal was distributed throughout the processes (Figure 1A) and was sometimes colocalized with receptor puncta (Figure 1B). The intensity of GABA immunoreactivity in the cell soma of neurons expressing the receptor was uniformly lower than that observed in the processes (Figure 1A).

We next determined whether GABA transaminase (GABA-T), a mitochondrial enzyme that degrades GABA, was present in neurons expressing surface GABA_A_ receptors. GABA-T immunoreactivity was observed in β2/3 subunit immunopositive neurons, where it was restricted to the cell soma (Figure 1C). The selective presence of GABA-T in the soma corresponds to the subcellular region in which GABA immunoreactivity was noted to be lowest.

### Inhibition of GABA Transaminase Decreases the Association between the GABA_A_ Receptor α1 Subunit and the ER Quality Control Protein Calnexin

Calnexin is an ER chaperone that binds to nascent proteins as they undergo repeated folding/unfolding cycles until native folding is achieved or terminal misfolding occurs (Araki and Nagata, 2011). Because pharmacological chaperones facilitate the release of their target proteins from the calnexin binding cycle (Morello and Bichet, 2001; Fan et al., 2005; Robert et al., 2005; Gong et al., 2006) and since calnexin is known to interact with GABA_A_ receptor subunits (Connolly et al., 1996; Bradley et al., 2008), we reasoned that if GABA acts as a ligand chaperone then manipulations that increase intracellular GABA levels should cause the release of GABA_A_ receptor subunits from the calnexin quality control system.

To examine this possibility, vigabatrin (i.e., gamma-vinyl-GABA, Sabril®), an irreversible inhibitor of GABA-T (Jung et al., 1977) that increases GABA levels both *in vivo* (Löscher and Hörstermann, 1994; Manor et al., 1996; Todd and Baker, 2008; Tong et al., 2009) and in neuronal cultures (Gram et al., 1988; Sonnewald et al., 2006) was used to elevate intracellular GABA levels. Neuronal cultures were treated with vigabatrin (0.5 μg/μl, 3 h) and the receptor α1 subunit was then immunoprecipitated using an anti-α1 subunit antibody. An anti-α1 subunit antibody was used for the immunoprecipitation experiments since we do not have an anti-β2/3 subunit antibody that is suitable for immunoprecipitation experiments and the α1 subunit is abundant in cortex (Pirker et al., 2000). Both cell lysates and immunoprecipitates were subjected to SDS PAGE/Western blotting using anti-α1 subunit and anti-calnexin antibodies.

**Figure 2:**
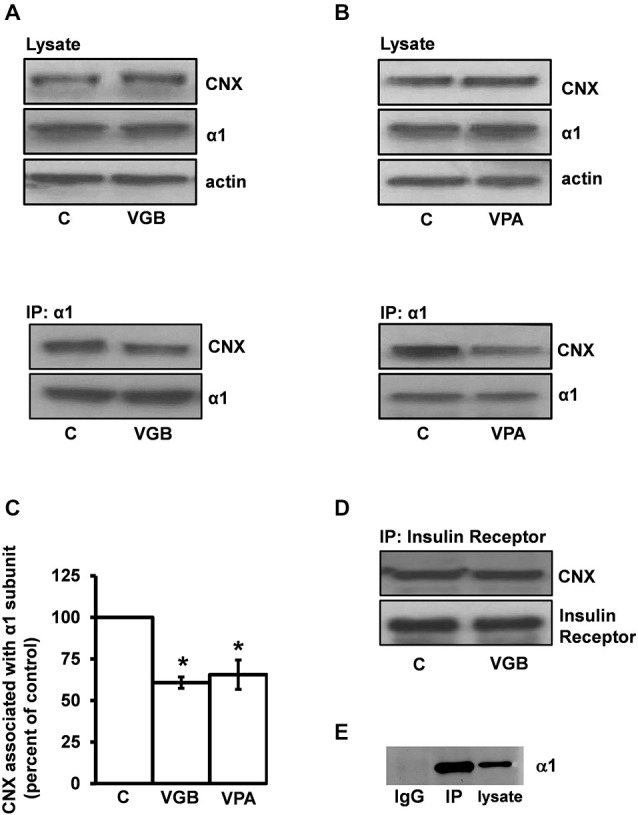
**Inhibition of GABA transaminase decreases the association of the GABA_A_ receptor α1 subunit with the ER quality control protein calnexin. (A)** High-density neuronal cultures were treated with vigabatrin (VGB) (0.5 μg/μl, 37°C) for 3 h. Neurons were then lysed and the α1 subunit was immunoprecipitated under nondenaturing conditions with an anti-α1 subunit antibody. Cell lysates and immunoprecipitated samples were subjected to SDS PAGE/Western blotting using GABA_A_ receptor anti-α1 subunit and anti-calnexin antibodies as indicated. Shown is a representative immunoblot. **(B)** Neurons were treated with valproic acid (VPA) (200 μM, 37°C) for 3 h and processed as described in **(A)**. Shown is a representative immunoblot. **(C)** Replicate data for immunoprecipitated samples described in **(A)** and **(B)**. Immunoreactive bands were quantified densitometrically. Data are presented as the average ± SEM. *p ≤ 0.05, paired *t*-test, *n* ≥ 3. Experiments *A* and *B* were conducted on cultures made from different litters and *t*-tests were performed on their respective controls. **(D)** Neurons were treated with VGB as described in **(A)** and cell lysates were subjected to immunoprecipitation with an anti-insulin receptor β chain antibody. Immunoprecipitates were resolved by SDS PAGE and visualized by Western blotting with anti-insulin receptor β chain and anti-calnexin antibodies. The blot shown is representative of three independent experiments. **(E)** Immunoblotting with an anti-α1 subunit antibody followed by a light-chain specific secondary antibody was performed on neuronal lysates and samples immunoprecipitated with either an anti-α1 subunit antibody or control IgG. No band is detected in the IgG control lane demonstrating that the IgG light-chain specific secondary antibody does not recognize the 50 kDa IgG heavy-chain.

Vigabatrin treatment significantly decreased the amount of calnexin co-immunoprecipitated by the anti-α1 subunit antibody to 60.8 ± 3.4% (average ± SEM) of vehicle-treated controls (*p* ≤ 0.0005, paired *t*-test, *n* = 5) (Figures 2A,C). Importantly, vigabatrin treatment did not affect the amount of α1-subunit immunoprecipitated (126.1 ± 23.8% of control) or the amounts of either the α1-subunit (107.9 ± 6.6% of control) or calnexin (107.1 ± 7.1%) in total cell lysates. We next tested a second GABA-T inhibitor, the anticonvulsant valproic acid (Depakote®) (Figures 2B,C). Treatment of the neurons with valproic acid (200 μM, 3 h) decreased the amount of calnexin coimmunoprecipitated by the anti-α1 subunit antibody to 65.5 ± 8.8% (average ± SEM) of control (*p* ≤ 0.05, paired *t*-test, *n* = 3). Similar to vigabatrin, valproic acid treatment did not affect the amount of α1-subunit that was immunoprecipitated (91.4 ± 3.8%). Since both GABA-T inhibitors produced similar results, it is likely that their ability to decrease the α1 subunit/calnexin interaction is due to the inhibition of GABA-T rather than to off-target effects such as histone deacetylase inhibition by VPA (Eyal et al., 2004).

To control for non-specific effects that GABA-T inhibition may have on secretory pathway processes, negative control experiments were performed. The insulin receptor was used for this purpose since it is presumed to be present in GABA_A_ receptor-containing neurons (Wan et al., 1997) and is known to interact with calnexin (Bass et al., 1998). Following vigabatrin treatment, the amount of calnexin immunoprecipitated by an anti-insulin receptor β chain antibody was 110.5 ± 13.6 (average ± SEM) of the control, *n* = 3 (Figure 2D, representative blot) which was not significantly different from control indicating that inhibition of GABA-T does not cause global disruption of the calnexin quality control system.

### Vigabatrin Treatment Promotes Forward-Trafficking of the GABA_A_ Receptor through the Secretory Pathway

The ability of GABA-T inhibitors to decrease the interaction between the receptor α1 subunit and calnexin suggests that an elevation in intracellular GABA levels may facilitate receptor maturation and cause the release of conformationally mature GABA_A_ receptors from the ER quality mechanisms. If so, there should be a corresponding increase in the forward-trafficking of the receptor through the secretory pathway.

As glycoproteins undergo forward-trafficking through the secretory pathway there are compartment-specific modifications made to their N-linked glycans that can be used to monitor anterograde glycoprotein trafficking (Freeze, 2001). *N*-linked glycosylated proteins within the ER contain high-mannose glycans (immature) that are sensitive to digest by the Endoglycosidase H (Endo H), while those in or beyond the trans-Golgi contain low-mannose glycans (mature) that are resistant to Endo-H digest. Thus, Endo H digest assays can be used to distinguish immature glycoproteins in the early secretory pathway from mature glycoproteins.

**Figure 3:**
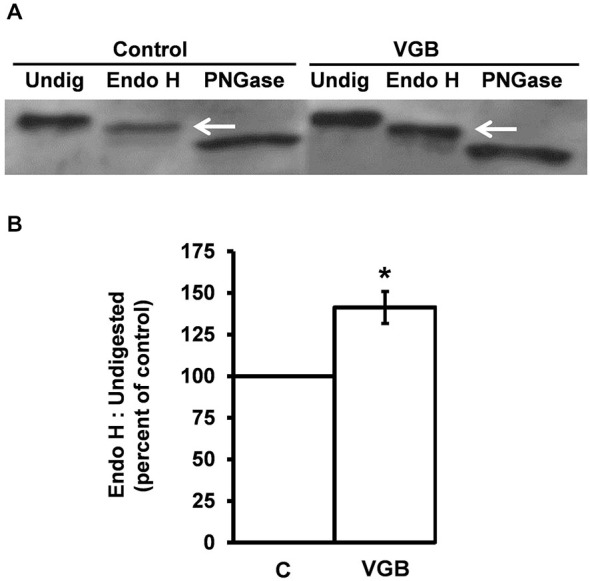
**Vigabatrin treatment promotes forward-trafficking of GABA_A_ receptors through the secretory pathway**. High-density neurons were incubated in the absence or presence of vigabatrin (VGB) (0.5 μg/μl, 37°C) for 6 h. The GABA_A_ receptor α1 subunit was then immunoprecipitated from neuronal lysates. Immunoprecipitated samples from each condition were then split into three equal samples and digested with either Endo H or PNGase or left undigested. Samples were resolved by SDS PAGE and analyzed by Western blotting using an anti-α1 subunit antibody. **(A)** A representative Western blot. Undigested samples yielded a single band at 50 kDa. Digest with PNGase, which removes all N-linked glycans, resulted in a 46 kDa band. Endo H digest produced a 48 kDa band, indicating that the α1 subunit is partially resistant to Endo H and, therefore, represents a mature (post ER) form of the α1 subunit. **(B)** Replicate data for immunoblot experiments described in **(A)**. Data shown are the ratio of the Endo H resistant band to the undigested band presented as average ± SEM. *p ≤ 0.05, paired *t*-test, *n* ≥ 4.

The GABA_A_ receptor α1 subunit contains two N-linked glycans (Chen et al., 2012). We first examined the glycosidase sensitivity of the α1 subunit. Neuronal cultures were lysed and the α1 subunit was immunoprecipitated with an anti-α1 subunit antibody. Immunoprecipitated samples were then digested with either Endo H or N-glycosidase F (PNGase), a glycosidase that cleaves N-linked glycans from both immature and mature glycoproteins thus yielding complete N-linked deglycosylation. Following glycosidase digest, samples were subjected to SDS PAGE and Western blotting with an anti-α1 subunit antibody. As expected, a Western blot of undigested α1 subunit resulted in a band that migrated at a molecular mass of 50 kDa (Figure 3A). Digest of immunoprecipitated samples with PNGase resulted in a single band of approximately 46 kDa, consistent with the complete removal of two N-linked glycans, each approximately 2 kDa. Endo H digest resulted in a band at approximately 48 kDa, indicating that the α1 subunit is only partially sensitive to Endo H digest. Partial digest can occur when a protein contains multiple N-linked glycans and one (or more) high-mannose glycan is sterically protected from processing in the secretory pathway, thus resulting in a mature protein with both Endo H-sensitive and Endo H-resistant glycans, as is the case for the GABA_A_ receptor β2 subunit (Lo et al., 2010). Curiously, no “immature” bands were observed. One possible explanation for this results is that all the α1 subunit is “mature”. This, however, is not the case since the ER quality control protein is abundantly coimmunoprecipitated by an anti-α1 subunit antibody (Figure 2). At this time we do not have an explanation for the absence of an “immature” band but note that a similar outcome in primary neuronal cultures has been reported (Nair et al., 2013).

Because the 48 kDa band represents α1 subunits that have acquired Golgi-mediated partial Endo H-resistance, the 48 kDa band is the mature form of the α1 subunit. To determine if treatment with a GABA-T inhibitor increases the forward-trafficking of GABA_A_ receptors, neurons were incubated in the absence (control) or presence of vigabatrin for 6 h. This treatment duration was chosen to allow sufficient time for chaperoned receptors to reach the trans-Golgi compartment. Following drug treatment, the neurons were lysed, the α1 subunit was immunoprecipitated and immunoprecipitates were either undigested or digested (Endo H or PNGase) and samples were then subjected to SDS PAGE/Western blotting (Figure 3A). To determine whether vigabatrin treatment resulted in an increase in the amount of mature α1 subunit, a ratio of mature (48 kDa) to total undigested (50 kDa) α1 subunit was calculated (Lo et al., 2010). Vigabatrin treatment increased the amount of mature α1 subunit to 141 ± 9.6% (average ± SEM) of that observed in vehicle-treated control neurons (*p* ≤ 0.05, paired *t*-test) (Figure 3B).

### Vigabatrin Treatment Increases Membrane Insertion of the Receptor

**Figure 4:**
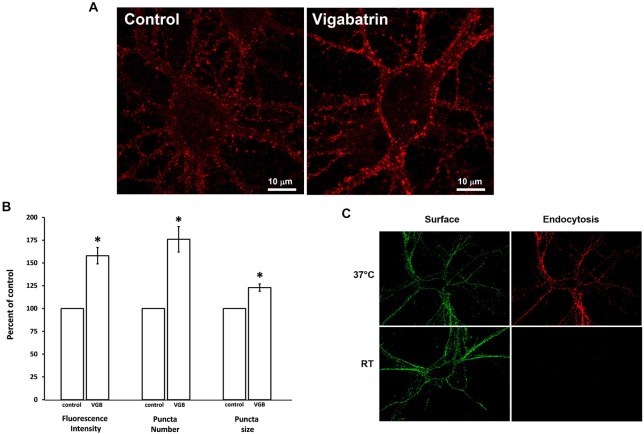
**Plasma membrane insertion of GABA_A_ receptors is increased by vigabatrin treatment**. Low-density cultures were incubated in the absence (control) or presence of VGB (0.5 mg/ml, 37°C) for 18 h. Anti-β2/3 subunit antibody immunofluorescence labeling in living cells was performed at room temperature (permissive for receptor insertion but not for endocytosis). Immunofluorescence was visualized by confocal microscopy. A Z-series of ten optical sections, each 0.2 μm thick, was collected and a 3D reconstruction was rendered. **(A)** Representative image.** (B)** Immunofluorescence intensity, puncta size and puncta number was quantified by an investigator blinded to the experimental conditions. Data shown are average ± SEM, *n* = 7. Comparisons between control and VGB treatment groups were made using paired *t*-tests corrected for multiple (3) comparisons *p ≤ 0.01. **(C)** Control experiment showing that receptor endocytosis occurs at 37°C but not room temperature. Living neurons were incubated in anti-β2/3 subunit antibody at either room temperature or 37°C to measure receptor endocytosis. After this incubation period the surface receptor population was labeled with Alexa 488-conjugated secondary antibody (green). Cells were then fixed, permeabilized and the endocytosed receptors were labeled with Alexa 594-conjugated secondary antibody (red). The brightness and contrast settings of images for receptor endocytosis (red) were enhanced to show that not even a faint signal is detectable at room temperature.

We next determined if vigabatrin treatment would increase the plasma membrane expression of the receptor. Neurons were treated with vigabatrin for 16 h and immunofluorescence labeling of surface receptors was performed using an anti-β2/3 subunit antibody. Live-cell immunofluorescence labeling was conducted at room temperature since room temperature is permissive for receptor insertion (Lu et al., 2001; Sun et al., 2005) but not endocytosis (Machu et al., 2006) and Figure 4C. This was important since vigabatrin treatment might promote GABA-induced GABA_A_ receptor endocytosis (Barnes, 1996), potentially masking increases in receptor membrane insertion if only cell surface expression was measured. Vigabatrin treatment significantly increased the fluorescence intensity, number of puncta and puncta size of surface receptors to 157 ± 9%, 176 ± 14%, and 123 ± 4% of control, respectively (average ± SEM, *n* = 7, *p* ≤ 0.01, paired *t*-test corrected for multiple comparisons) (Figure 4).

### The Ability of Vigabatrin to Decrease the Association of the Receptor α1 Subunit with Calnexin is not due to the Activation of Surface GABA_A_ or GABA_B_ Receptors

GABA-T inhibitors increase presynaptic GABA levels resulting in greater synaptic GABA release, elevated synaptic GABA concentrations, and, therefore, increased activation of surface GABA_A_ and GABA_B_ receptors (Wu et al., 2001, 2003, 2007; Peng et al., 2010). Thus, it was possible that the ability of vigabatrin to decrease the interaction between the GABA_A_ receptor α1 subunit and calnexin was due downstream consequences of surface GABA_A_ or GABA_B_ receptor activation.

**Figure 5:**
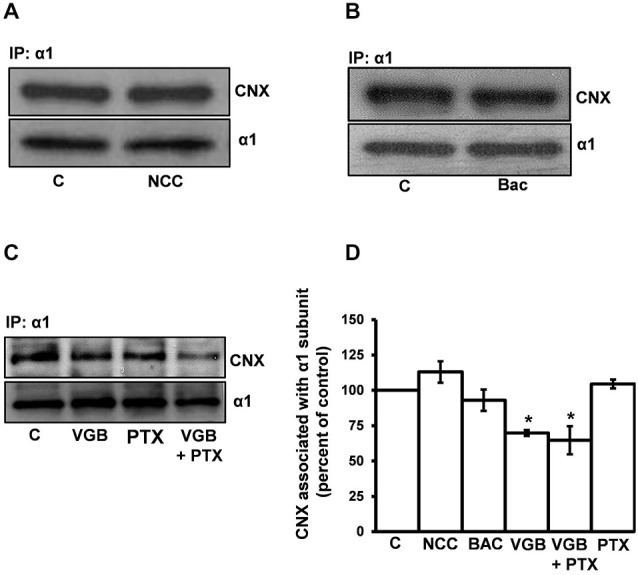
**The ability of vigabatrin treatment to decrease the association of the receptor α1 subunit with calnexin is not due to the activation of surface GABA_A_ or GABA_B_ receptors**. High-density neuronal cultures were treated for 3 h at 37°C with **(A)** DMSO (control) or the GAT blocker NCC 05-2090 (100 μM), **(B)** vehicle (control) or the GABA_B_ receptor agonist baclofen (100 μM), or **(C)** vehicle (control), VGB (0.5 μg/μl), VGB (0.5 μg/μl) + the GABA_A_ receptor channel blocker PTX (100 μM) or PTX (100 μM). Neuronal lysates were immunoprecipitated with an anti-α1 subunit antibody and SDS PAGE/Western blotting was performed with anti-α1 subunit and anti-calnexin antibodies. Representative blots are shown in **(A–C)**. **(D)** Replicate data (*n* ≥ 3) of experiments in **(A–C)**. Neuronal cultures from different litters were used for experiments in **(A–C)**. Data for each experiment were normalized to their respective controls and assembled into one graph. Immunoreactive bands were quantified densitometrically and data are presented as the average ± SEM. A one-way ANOVA performed on the data set from experiments in *C* yielded a *p* value of 0.0013. *Post hoc* significance differences (Tukey’s multiple comparison) between C vs. VGB and C vs. VGB + PTX were observed (*p ≤ 0.05). No statistically significant difference was observed between VGB vs. VGB + PTX treatment groups.

Synaptic levels of GABA are regulated by the GABA transporter GAT1, which transports GABA back into presynaptic neurons following synaptic GABA release (Madsen et al., 2010). Blockade of GAT1 results in an increase in synaptic GABA levels (Kersanté et al., 2013) and, thus, the activation of GABA_A_ and GABA_B_ receptor subtypes (Bragina et al., 2008; Gonzalez-Burgos, 2010). We reasoned that if the vigabatrin-mediated decrease in the α1 subunit/calnexin interaction was due to the activation of surface GABA_A_ or GABA_B_ receptors, then the inhibition of GAT1 should mimic the effect of vigabatrin. To test this, neurons were treated for 3 h with either DMSO (control) or NCC 05-2090 (100 μM), a non-selective GAT blocker at the concentration used here (Madsen et al., 2010). Immunoprecipitations were then conducted with the anti-α1 subunit antibody and SDS PAGE/Western blotting was performed (Figures 5A,D). The amount of calnexin coimmunoprecipitated by the anti-α1 subunit antibody in the NCC-treated neurons was 113.0 ± 7.6% (average ± SEM, *n* = 4) of vehicle-treated controls. Thus, a treatment known to elevate synaptic GABA levels and activate surface GABA_A_ and GABA_B_ receptors does not mimic the effects of vigabatrin.

We next determined whether the GABA_B_ receptor agonist baclofen would mimic the effects of vigabatrin (Figures 5B,D). Following baclofen treatment, the amount of calnexin immunoprecipitated by the α1 subunit was 93 ± 7.6% of control (average ± SEM, *n* = 3) indicating that activation of GABA_B_ receptors does not mimic the effect of vigabatrin on the α1 subunit/calnexin interaction. Baclofen treatment did not affect the amount of immunoprecipitated α1-subunit which was 97 ± 7.6% (average ± SEM) of control.

The involvement of surface GABA_A_ receptors was next tested. For these experiments, we chose not to use GABA_A_ receptor antagonists that bind to the GABA binding site since they could potentially act as GABA_A_ receptor pharmacological chaperones (Eshaq et al., 2010). Instead, we determined whether the GABA_A_ receptor channel blocker picrotoxin (PTX) could block the vigabatrin-mediated decrease in the α1 subunit/calnexin association. In these experiments PTX failed to block the vigabatrin effect (Figures 5C,D). Treatment with vigabatrin in the absence or presence of PTX significantly decreased the amount of calnexin coimmunoprecipitated by the anti-α1 antibody to 69 ± 2.1% and 64.7 ± 10%, respectively (average ± SEM) compared to vehicle-treated controls (one-way ANOVA followed by *post hoc* Tukey’s Multiple Comparison Test, *p* ≤ 0.05, *n* = 3). No significant difference was observed between vigabatrin and vigabatrin + PTX treatments. PTX treatment alone did not affect the α1 subunit/calnexin interaction (104.5 ± 3.2% of control, average ± SEM, *n* = 3). Vigabatrin, PTX or PTX + vigabatrin treatments did not affect the amount of α1-subunit immunoprecipitated (102.9 ± 8.5%, 101.1 ± 18.5%, 101.3 ± 25.4%, average ± SEM, of control, respectively). Collectively, these data indicate that the ability of the GABA-T inhibitor vigabatrin to decrease the association of the α1 subunit with calnexin is not a downstream consequence of surface GABA_A_ or GABA_B_ receptor activation.

### The Ability of Vigabatrin to Decrease the Interaction between the Receptor α1 Subunit and Calnexin is Prevented by the GAT Inhibitor NCC 05-2090

**Figure 6:**
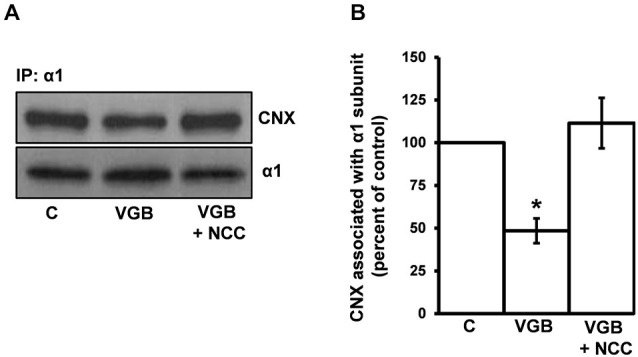
**The GAT inhibitor NCC 05-2090 prevents the vigabatrin-mediated decreases in the association of the receptor α1 subunit and calnexin**. High-density neuronal cultures were treated with vehicle (control), VGB (0.5 μg/μl), or VGB (0.5 μg/μl) + NCC 05-2090 (100 μm) at 37°C for 3 h. Neuronal lysates were immunoprecipitated with an anti-α1 subunit antibody and immunoprecipitates were subjected to SDS PAGE/Western blotting with anti-α1 subunit and anti-calnexin antibodies. **(A)** A representative blot. **(B)** Replicate data for experiments described in **(A)**. Immunoreactive bands were quantified densitometrically and data plotted. Data are presented as the average ± SEM. Using a one-way ANOVA followed by a Tukey’s Multiple Comparison Test, VGB treatment was significantly different from both control and VGB + NCC conditions *p ≤ 0.01, *n* ≥ 4. No significant difference between control and VGB + NCC conditions was observed.

For GABA to act as a ligand chaperone in the RER, GABA must gain access to the RER lumen. While it is not clear how this occurs, GABA, a zwitterionic amino acid, may require active transport to enter the RER by either a GAT or GAT-like transporter. To determine if such transport might be involved in the vigabatrin effect, we tested whether the GAT blocker NCC 05-2090 (100 μM) could block the vigabatrin effect. Vigabatrin and vigabatrin + NCC treatments altered the amount of calnexin immunoprecipitated by the anti-α1 subunit antibody to 48.5 ± 7.3% and 111.5 ± 14.7% of control, respectively (average ± SEM, *n* = 4) (Figure 6). These values were significantly different from each other (one-way ANOVA, followed by *post hoc* Tukey’s Multiple Comparison Test, *p* ≤ 0.001). The vigabatrin + NCC treatment group was not significantly different from control. Thus, treatment with a GAT blocker prevents the vigabatrin-mediated decrease in the α1 subunit/calnexin interaction. Neither vigabatrin nor vigabatrin + NCC treatment significantly affected the amount of α1-subunit that was immunoprecipitated (129 ± 17.5% and 96 ± 3.1%, average ± SEM of control, respectively).

### GABA is Present within the RER Lumen

**Figure 7:**
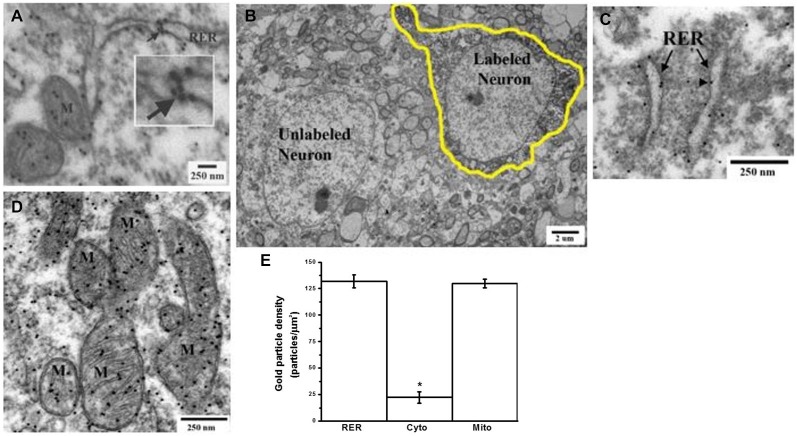
**Electron microscopy immunogold-labeling demonstrates the presence of GABA within the lumen of the endoplasmic reticulum of glutamatergic cortical neurons. (A)** Postembedding immunogold-labeling of GABA in slices of mouse primary motor cortex neurons shows GABA present within the lumen of the rough endoplasmic reticulum (RER) in a neuron of unidentified phenotype. Intraluminal and outer RER membrane associated GABA labeling are indicated by arrow. Inset is a higher magnification of a cluster of GABA immunogold-labeling within the RER lumen. Mitochondria (M) displaying dense GABA labeling. **(B)** Identification of a glutamatergic neuron of the motor cortex (hand-drawn outline) by retrograde tracing with biotinylated dextran amine (BDA) injected into the dorsolateral striatum. **(C)** Representative BDA-labeled glutamatergic neuron showing postembedding immunogold GABA labeling of the RER in the cell soma **(D)** Representative BDA-labeled glutamatergic neuron showing postembedding immunogold GABA labeling of mitochondria (M) in the cell soma. **(E)** Replicate data from multiple tissue sections from BDA-labeled glutamate neurons analyzed for the density of immunogold GABA labeling of various subcellular compartments (average ± SEM, *n* ≥ 31). Gold particle densities in the RER and mitochondria were each significantly greater than that in cytoplasm (*Tukey-Kramer HSD, *p* ≤ 0.0001).

Because the GABA chaperoning hypothesis depends on the presence of GABA in the RER lumen, we next determined whether GABA could be detected within the RER of neurons. For these experiments, mouse brain slices were immunogold-labeled using an anti-GABA antibody. Gold particles were then detected by electron microscopy (Figure 7A). Consistent with the known subcellular distribution of GABA (Belenky et al., 2003), immunogold-labeling was detected in the cytoplasm and mitochondria, where GABA is synthesized and degraded, respectively (Pinal and Tobin, 1998). Importantly, GABA labeling was also observed within the lumen of the RER, as well in association with the outer leaflet of the RER membrane. Of particular note, a cluster of immunogold particles within the RER was observed (Figure 7A inset).

### GABA is Present within the RER Lumen of Glutamatergic Pyramidal Neurons in the Motor Cortex

Our initial EM experiments did not identify the type of neuron (i.e., GABAergic interneuron, glutamatergic pyramidal neuron, cholinergic etc.) in which GABA was detected in the RER. Since GABA_A_ receptors are largely postsynaptic (Luscher et al., 2011) and present on glutamatergic neurons (Nusser et al., 1996, 1998; Nyíri et al., 2001; Brickley and Mody, 2012), we next determined if GABA could be found within the RER of glutamatergic neurons of the motor cortex. These neurons were identified using retrograde tracing by the injection of biotinylated dextran amine (BDA) into the dorsolateral striatum (Figure 7B). Glutamatergic motor cortex neurons displayed immunogold GABA labeling associated with both the outer face of the RER membrane and within the RER lumen, as well as within mitochondria and cytoplasm (Figures 7C,D). The densities of GABA labeling of the RER, cytoplasm and mitochondria were 131.8 ± 6.2, 22.3 ± 5.6, and 129.8 ± 4.2 particles/μm^2^ (average ± SEM, *n* ≥ 31), respectively (Figure 7E). These densities were significantly different (ANOVA, *p* ≤ 0.0001). Gold particle densities in the RER and mitochondria were each significantly greater than that in cytoplasm (Tukey-Kramer HSD, *p* ≤ 0.0001). The densities of GABA labeling in the RER and mitochondria were not significantly different from each other, suggesting that GABA is present in these two organelles at similar concentrations.

## Discussion

Previously we demonstrated that the neurotransmitter GABA acts as a ligand chaperone in the early secretary pathway to promote the biogenesis of recombinant GABA_A_ receptors (Eshaq et al., 2010). In the present study we extend these findings using primary neuronal cultures to show that endogenous GABA acts as a cognate ligand chaperone for neuronal GABA_A_ receptors. Evidence in support of this conclusion includes the presence of both GABA and GABA-T in GABA_A_ receptor-containing neurons and the observation that drug treatments (GABA-T inhibitors) that elevate intracellular GABA levels decrease the interaction of the receptor with the RER quality control protein calnexin, and subsequently promote the forward-trafficking and membrane insertion of the receptor. The effect of GABA-T inhibitor treatment is neither due to the activation of surface GABA_A/B_ receptors nor is it a general effect on secretory system processing. Lastly, immunogold detection of GABA in the RER lumen provides key evidence in support of the chaperoning hypothesis.

For GABA to act as a chaperone for GABA_A_ receptors, GABA must be present in neurons that synthesize the receptor. In our primary cortical cultures, approximately 55% of the GABA_A_ receptor-positive neurons were observed to possess GABA immunofluorescence labeling. Since only 20–30% of cortical neurons are thought to be GABAergic (Markram et al., 2004) and we have not intentionally enriched our cultures for interneurons, it is likely that non-GABAergic neurons in our culture contain GABA as is known to occur in cerebellar neuronal cultures (Suñol et al., 2010). We suggest that chaperoning may occur in neurons of various phenotypes (GABAergic interneurons, glutamatergic pyramidal neurons, etc.). Such a notion is consistent with our ability to detect chaperoning by biochemical methods. Although we have not yet characterized the type of neurons in which chaperoning occurs, our study shows that GABA is present in the RER of glutamatergic pyramidal cells, a neuron type rich in postsynaptic GABA_A_ receptors (Nusser et al., 1996, 1998; Nyíri et al., 2001; Brickley and Mody, 2012). The idea that GABA may chaperone GABA_A_ receptors in glutamatergic neurons may seem unexpected, however, others have noted that glutamatergic granule cells contain GABA, GAD67 and GABA transporters (Ramírez and Gutiérrez, 2001; Bergersen et al., 2003; Sirvanci et al., 2005; Suñol et al., 2010; Sperk et al., 2012; Ruiz and Kullmann, 2013; Root et al., 2014) and it is possible that other types of glutamatergic neurons may also contain GABA and GABAergic proteins.

Calnexin is an ER resident lectin that interacts with monoglucosylated N-linked glycans within nascent glycoproteins to aid in their folding (Araki and Nagata, 2011). Calnexin quality control is a dynamic process whereby protein intermediates undergo multiple binding/unbinding cycles until either a native structure is attained or proteins are targeted for degradation. It is well established that pharmacological chaperones facilitate the release of their target proteins from the calnexin binding/unbinding cycle and that this release is accompanied by an increase in forward-trafficking and membrane insertion (Morello and Bichet, 2001; Fan et al., 2005; Robert et al., 2005; Gong et al., 2006). Similarly, we show that increases in intracellular GABA levels (via GABA-T inhibition) decrease the association of GABA_A_ receptor α1 subunit with calnexin while promoting receptor forward-trafficking and membrane insertion. This effect is not due to a general effect on secretory pathway processing since inhibition of GABA-T does not affect the association of calnexin with the insulin receptor β chain. Furthermore, the effect is not due to the activation of surface GABA_A_ or GABA_B_ receptors since it is neither mimicked by the activation of surface GABA receptors nor blocked by the GABA_A_ receptor channel blocker picrotoxin.

Since the topology of the GABA_A_ receptor places the GABA binding sites within the RER lumen, GABA must access the RER lumen to act as a cognate ligand chaperone. Our electron microscopy immunogold-labeling experiments in cortical slices show that GABA is present both within the RER lumen and associated with the outer face of the RER membrane. This finding is consistent with GABA labeling over the Golgi apparatus of rat suprachiasmatic nucleus neurons (Belenky et al., 2008), and, although not described by the authors, RER luminal GABA labeling (Figure 13 in Belenky et al. (2008)). Interestingly, electron microscopy immunogold-labeling experiments show that not only is GABA present within the RER lumen of pancreatic β cells, but that RER GABA levels are dynamically regulated by drug treatment in an organelle-specific manner (González del Pliego et al., 2001). Such findings, coupled with those of the present study, support a physiological role for GABA in the RER lumen. Whether GABA and GABA_A_ receptors are colocalized in the RER of neurons remains to be determined, as does whether manipulation of GABA levels by GABA-T inhibitors affects GABA distribution in the RER.

While our experiments do not measure the absolute concentration of GABA in the RER, intracellular GABA concentrations have been estimated to be in the low mM range (Otsuka et al., 1971; Rothman et al., 1993; Wu et al., 2007). Our electron microscopy data show that the density of GABA immunogold labeling in the RER and mitochondria is approximately six times that of the cytoplasm. This suggests that GABA concentrations in the RER are likely to be sufficient for chaperoning to occur since the GABA EC_50s_ for GABA_A_ receptors are in the low μM range. We should point out, however, that the GABA binding affinity of nascent receptors (α/β dimers or higher order oligomers) may different from that of surface receptors. Furthermore, it is not clear how the gel-like matrix of the RER intraluminal environment may affect GABA binding.

An important unanswered question is how GABA accesses the ER lumen. While it is possible that GABA may enter the ER through open translocons following ribosomal dissociation (Lizák et al., 2008) or via passive diffusion through a “leaky” ER membrane (Le Gall et al., 2004), we consider this unlikely since the density of GABA labeling in the RER is approximately six times that of the cytoplasm. Because the density of GABA labeling in the RER is similar to that in mitochondria, an organelle for which GABA is actively transported (Passarella et al., 1984; Berkich et al., 2007), we suggest that GABA is actively transported into the RER. Such transport would potentially provide control over intraluminal GABA concentration. It is conceivable that the high density cluster of GABA labeling that we observed in the RER may represent a site of specific uptake.

It is possible that GABA may be actively transported into the ER by one of the four known high-affinity GABA transporters (GAT1–3 and BGT1) (Madsen et al., 2010; Zhou and Danbolt, 2013). Although we do not know if GATs can transport GABA into the RER, our data show that the vigabatrin-mediated decrease in the α1 subunit/CNX interaction is blocked by a GAT inhibitor used at a concentration that inhibits all GAT isoforms. As integral membrane proteins, GATs are at least transiently present in the RER and might function during their RER residency, as is the case for glucose transporters (Takanaga and Frommer, 2010). Additionally, GATs in isolated RER membrane vesicles exhibit functional properties similar to GATs at the plasma membrane (Scholze et al., 2002). Furthermore, the intracellular concentrations of sodium and GABA, as well as the estimated ER membrane potential, appear to offer a favorable driving force for GAT-mediated reverse transport of GABA into the ER (Eshaq et al., 2010). Based on the cell-type specific distributions of GAT isoforms and the heterogeneity of cell-types expressing the various GABA_A_ receptor subtypes (Madsen et al., 2010; Suñol et al., 2010; Schousboe et al., 2013), it is conceivable that different GAT isoforms could reverse transport GABA into the RER depending on cell-type.

Alternatively, a non-GAT mechanism may transport GABA into the RER. Because little is known regarding transporters that maintain the RER environment (Csala et al., 2007; Takanaga and Frommer, 2010), any attempts to identify a putative RER GABA transporter will likely be challenging. In this regard, it has been recognized for many decades that GABA enters mitochondria to be degraded by GABA-T. Surprisingly, however, the identity of the mitochondrial GABA transporter remains elusive despite long-standing evidence of its existence (Passarella et al., 1984; Berkich et al., 2007) and the identification of numerous mitochondrial SLC25 family members (Gutiérrez-Aguilar and Baines, 2013; Rudnick et al., 2014). Transport of GABA into the mitochondria of the plant *Arabidopsis thaliana* has recently been demonstrated to be mediated by AtGABP (Michaeli et al., 2011), a GABA permease with sequence homology to the amino acid-polyamine-organocation (APC) superfamily. Other GABA permeases, transporters or carriers include the glutamate/GABA antiporter in E. Coli used for acid resistance (Ma et al., 2013) and three GABA permeases/transporters in *Saccharomyces cerevisiae* (Kamei et al., 2011; Cao et al., 2013). A basic local alignment search tool (BLAST) analysis reveals that none of the above mentioned GABA carriers/transporters are homologous to GATs. Each, however, shares homology with the L-type amino acid transporter (LAT2), the light subunit for 4F2hc. LAT2 is a neutral/zwitterionic amino acid transporter whose mRNA and protein is present in many tissues including brain (Pineda et al., 1999; Cajigas et al., 2012; Zielińska et al., 2014). The substrate K_ms_ of LAT2 are in the micromolar range, well within the affinity range needed to transport cytoplasmic GABA which is present at low millimolar intracellular concentrations within both neurons (Otsuka et al., 1971; Rothman et al., 1993; Wu et al., 2007) and astrocytes (Lee et al., 2011).

The molecular mechanism by which GABA acts as a ligand chaperone remains to be determined. Pharmacological chaperones have been observed to stabilize protein structure, release mature receptors from ER quality control mechanisms, promote oligomerization of multimeric proteins, and facilitate ER export (reviewed in Leidenheimer and Ryder, 2014). Because none of these mechanisms is mutually exclusive, all could be envisioned to apply to GABA chaperoning of GABA_A_ receptors as steps along a serial pathway of maturation and export. The GABA_A_ receptor is a heteropentamer with a stoichiometry of αβαβγ/δ subunits forming a central chloride pore (Luscher et al., 2011). Two orthosteric GABA binding sites are contained within the receptor, one at each αβsubunit interface (Amin and Weiss, 1993) with the minimum requirement for GABA binding to an αβ heterodimer. While it is not known whether the chaperoning effect of GABA promotes the assembly process or occurs following pentamerization, nicotine chaperoning of nicotinic acetylcholine receptors occurs prior to pentamerization and facilitates subsequent oligomerization steps (Srinivasan et al., 2011; Mazzo et al., 2013). In this light, we propose that GABA binds to an αβ heterodimer, or higher-order receptor intermediate, resulting in conformational changes that lower the energy barrier for further oligomerization steps. In support of this idea, key GABA binding residues on the α1 subunit (Boileau et al., 1999) are in close proximity to residues involved in receptor assembly (Taylor et al., 2000; Sarto et al., 2002; Bollan et al., 2003). Alternatively, GABA binding to pentameric receptors may produce structural alterations that release the receptor from ER quality control proteins and/or expose export signals. In any case, chaperoning does not require structural changes associated with agonist-induced channel-gating since the competitive antagonist (+)bicuculline is an effective pharmacological chaperone of GABA_A_ receptors (Eshaq et al., 2010). Regardless of molecular mechanism(s), it is likely that chaperoning is not required for receptor biogenesis, but merely serves a regulatory function.

In our immunofluorescence experiments we note some surface receptor puncta that are colocalized with GABA. While we did not further investigate this phenomenon, we offer several hypothetical explanations. One intriguing possibility is that chaperoned receptors may be inserted into the plasma membrane in a GABA-bound state. In this regard, growth hormone receptors that are chaperoned by growth hormone are inserted into the plasma membrane as desensitized receptor/ hormone complexes (van den Eijnden and Strous, 2007). For the GABA_A_ receptor to arrive at the surface as a liganded receptor, GABA would need to be either present throughout the secretory pathway to allow equilibrium binding/unbinding of GABA (we have observed GABA in the Golgi by immunogold labeling) or, alternatively, GABA might become “locked on” to the receptors in the secretory pathway, as is postulated to occur when surface receptors bind GABA (Khatri et al., 2009). For our experiments, the detection of such liganded receptors on the surface would require that GABA remain bound during receptor immunolabeling and subsequently be available for antibody recognition during GABA labeling, which, while possible, seems unlikely. More likely, due to the limited spatial resolution of confocal microscopy, the colocalized signal may represent receptor cell surface labeling that is in close proximity to GABA-containing structures that are just beneath the plasma membrane.

What might be the physiological significance of GABA acting as a chaperone for its receptor? Many integral membrane proteins are “inefficiently” processed in the RER with up to 70% of nascent subunits/receptors degraded without being used. These proteins include G protein-coupled receptors (gonadotropin releasing hormone, calcium-sensing, δopioid, and V2 vasopressin receptors), ion channels (nicotinic acetylcholine receptors, GABA_A_ receptors, Na_v_ sodium channels), and growth hormone receptors (Merlie and Lindstrom, 1983; Schmidt et al., 1985; Gorrie et al., 1997; Janovick et al., 2002; Petäjä-Repo et al., 2002; Wüller et al., 2004; Robert et al., 2005; Sallette et al., 2005; Huang and Breitwieser, 2007; van den Eijnden and Strous, 2007). Interestingly, many of these wild type subunits/receptors are not terminally misfolded since native folding, forwarding trafficking and function can be “rescued” by treatment with either pharmacological chaperones (Janovick et al., 2002; Petäjä-Repo et al., 2002; Wüller et al., 2004; Robert et al., 2005; Sallette et al., 2005; Huang and Breitwieser, 2007) or proteasome inhibitors (Christianson and Green, 2004). Thus, for inefficiently processed secretory pathway proteins, it has been suggested that the RER serves as a storage reservoir of viable folding intermediates that are subject to post-translational regulatory mechanisms (Leidenheimer and Ryder, 2014). Such a reservoir of newly-synthesized proteins could be accessed to rapidly increase functional pools without involving transcriptional or translational processes. For receptors in which a large percentage of viable proteins exist as an RER reserve pool, a modest recruitment from the RER pool would significantly increase the functional pool. While we have not measured GABA-gated chloride currents in our experiments here, we note that in HEK 293 cells, GABA chaperoning of recombinant receptors results in a concomitant increase in receptor function (Eshaq et al., 2010) and that increased cell surface expression of the receptor is highly correlated with larger peak amplitude GABA_A_ receptor responses (Vithlani et al., 2011).

In addition to regulating to the number of surface receptors, the chaperoning of GABA_A_ receptors may favor the production of certain GABA_A_ receptor subtypes, potentially controlling the balance of phasic:tonic GABAergic inhibition. Phasic inhibition is a fast, transitory process that is mediated by γsubunit-containing synaptic receptors that bind GABA with low-affinity. Tonic inhibition is a slow, sustained process mediated by δ subunit-containing extrasynaptic receptors that bind GABA with high-affinity (Farrant and Nusser, 2005; Belelli et al., 2009). These types of GABA_A_ receptor-mediated inhibition have unique roles in maintaining physiological processes and contributing to various pathophysiologies. We envision two possible mechanisms by which chaperoning could bias the production of receptor subtypes that participate in these two distinct modes of inhibition. First, GABA binding could favor the incorporation of either the γ or δ subunit into the pentamer during the assembly process. In this regard, nicotine acting as a pharmacological chaperone prior to nicotinic acetylcholine receptor pentamerization, biases the incorporation of a β3 subunit over an α subunit into the accessory fifth position, thus allowing for post-translational subunit switching (Srinivasan et al., 2011; Mazzo et al., 2013). Secondly, because GABA_A_ receptor subtypes have widely varying affinities for GABA (Farrant and Nusser, 2005), it is possible that tightly-controlled RER luminal GABA concentrations may favor the biogenesis of high-affinity extrasynaptic GABA_A_ receptors.

Where in the cell does chaperoning occur? The subcellular site of GABA_A_ receptor subunit synthesis and assembly is not yet known, however, it is presumed to occur in the soma as well as in dendrites (see below). While our experiments do not address where in the neuron chaperoning occurs, we note that GABA is present in the cell soma, albeit at apparently lower concentrations than in dendrites. Furthermore, our immunogold labeling detects GABA in somatic RER in glutamatergic neurons. With respect to dendrites, our data and those from previous immunogold-labeling experiments (Fujiyama et al., 2000), place postsynaptic GABA_A_ receptors and intracellular GABA in close proximity in dendrites, suggesting that chaperoning may occur in dendritic compartments. In this regard, dendrites possess a prodigious anastomosing ER (Horton and Ehlers, 2003) with over 2500 mRNAs identified in the hippocampal synaptic neuropil including an abundance of neurotransmitter receptor mRNAs (Cajigas et al., 2012). Indeed deep sequencing of the synaptic neuropil transcriptome (Cajigas et al., 2012) and laser capture microdissection of dendrites in rat motor cortex (Costa et al., 2002) have revealed the presence of eight distinct dendritically-localized GABA_A_ receptor subunit mRNAs. Because of the abundance of GABA in neuronal processes noted here and elsewhere (Fujiyama et al., 2000) and the presumed translation of GABA_A_ receptor subunit mRNAs in dendrites, we propose that GABA chaperoning targets dendritically synthesized receptors/receptor intermediates, perhaps in close proximity to inhibitory synapses.

The synthesis of GABA from its precursor glutamate occurs in the cell cytoplasm catalyzed by glutamic acid decarboxylase isoforms, GAD 65 and GAD 67 (Pinal and Tobin, 1998). GAD65 is localized primarily to presynaptic terminals and produces a “transmitter” pool of GABA. GAD 67 has a ubiquitous central nervous system distribution and is responsible for maintaining low millimolar concentrations of “metabolic” GABA in the cytosol (Otsuka et al., 1971; Rothman et al., 1993; Pinal and Tobin, 1998; Wu et al., 2007; Lee et al., 2011). Whether “chaperone” GABA is derived from “metabolic” and/or “transmitter” GABA pools remains to be determined, however, because of the widespread cellular distribution of “metabolic” GABA, it is likely that at least some “chaperone” GABA is derived from the metabolic pool, i.e., synthesized by GAD67. The source of “chaperone GABA” is important as it will aid in determining whether chaperoning is a cell autonomous or non-autonomous process. The ability of GABA to be redistributed between cells by both GAT and non-GAT transport mechanisms, into both GABAergic and non-GABAergic neurons, as well as astrocytes (Madsen et al., 2010; Suñol et al., 2010; Zhou and Danbolt, 2013) will complicate efforts to characterize the source of “chaperone” GABA.

While the physiological significance of “metabolic” GABA is not understood, it is worth noting that approximately ninety percent of GABA in newborn mice is synthesized via GAD67 (Asada et al., 1997), implying an important role for “metabolic” GABA and GAD67 in brain function. The observations that GAD67 protein and/or mRNA levels increase subsequent to sensory learning (Gierdalski et al., 2001), hippocampal kindling (Ramírez and Gutiérrez, 2001), voluntary exercise (Hill et al., 2010), ischemia (Li et al., 2010), and benzodiazepine discontinuation (Izzo et al., 2001) indicate that GABA levels in the brain are dynamically regulated in response to a wide range of stimuli. Such regulation could be envisioned to impact GABA chaperoning by controlling intracellular GABA concentrations. Tangentially, it has long been recognized that GABA synthesis and degradation are linked to the tricarboxylic acid cycle via the “GABA shunt”, a closed-loop circuit allowing for both the production of GABA and the conservation of its precursor glutamate (Martin, 1993). The relationship between the tricarboxylic acid cycle and GABA production may potentially link GABA chaperoning of GABA_A_ receptors to energy metabolism.

In conclusion, our findings provide evidence that neuronal GABA_A_ receptors undergo cognate ligand chaperoning in the RER by endogenous GABA. While many questions remain, we propose that the RER functions as a local reservoir of receptor intermediates that allows GABA chaperoning-dependent recruitment of nascent receptors into the functional pool. Such a mechanism may have evolved to allow rapid and local post-translational control over functional receptor pools in a morphologically complex cell. Because GABA is used as a neurotransmitter, paracrine signaling molecule, trophic factor and energy metabolite (Pinal and Tobin, 1998), its role as a cognate ligand chaperone may be uniquely complex.

## Contributors

NJL and CKM planned/designed the experiments. PW, RSE, CM and RLH performed the experiments. PW, RSE, NJL, CKM and CM analyzed the data. NJL and CKM wrote the manuscript.

## Conflict of Interest Statement

The authors declare that the research was conducted in the absence of any commercial or financial relationships that could be construed as a potential conflict of interest.
